# Management of chronic wasting disease in ranched elk: conclusions from a longitudinal three-year study

**DOI:** 10.1080/19336896.2020.1724754

**Published:** 2020-02-07

**Authors:** N.J. Haley, D.M. Henderson, R. Donner, S. Wyckoff, K. Merrett, J Tennant, E.A. Hoover, D. Love, E. Kline, A.D. Lehmkuhl, B.V. Thomsen

**Affiliations:** a Department of Microbiology and Immunology, College of Graduate Studies, Midwestern University, Glendale, AZ, USA; b Prion Research Center, Department of Microbiology, Immunology, and Pathology, College of Veterinary Medicine and Biomedical Sciences, Colorado State University, Fort Collins, CO, USA; c Colorado Department of Agriculture Animal Health Division, Broomfield, CO, USA; d National Veterinary Services Laboratories, United States Department of Agriculture, APHIS, VS, Ames, IA, USA; e Center for Veterinary Biologics, United States Department of Agriculture, APHIS, VS, Ames, IA, USA

**Keywords:** Prion, elk, RAMALT, RT-QuIC, antemortem

## Abstract

Chronic wasting disease is a fatal, horizontally transmissible prion disease of cervid species that has been reported in free-ranging and farmed animals in North America, Scandinavia, and Korea. Like other prion diseases, CWD susceptibility is partly dependent on the sequence of the prion protein encoded by the host’s *PRNP* gene; it is unknown if variations in *PRNP* have any meaningful effects on other aspects of health. Conventional diagnosis of CWD relies on ELISA or IHC testing of samples collected post-mortem, with recent efforts focused on antemortem testing approaches. We report on the conclusions of a study evaluating the role of antemortem testing of rectal biopsies collected from over 570 elk in a privately managed herd, and the results of both an amplification assay (RT-QuIC) and conventional IHC among animals with a several *PRNP* genotypes. Links between *PRNP* genotype and potential markers of evolutionary fitness, including pregnancy rates, body condition, and annual return rates were also examined. We found that the RT-QuIC assay identified significantly more CWD positive animals than conventional IHC across the course of the study, and was less affected by factors known to influence IHC sensitivity – including follicle count and PRNP genotype. We also found that several evolutionary markers of fitness were not adversely correlated with specific *PRNP* genotypes. While the financial burden of the disease in this herd was ultimately unsustainable for the herd owners, our scientific findings and the hurdles encountered will assist future CWD management strategies in both wild and farmed elk and deer.

## Introduction

Chronic wasting disease (CWD) is a naturally occurring, progressive and ultimately fatal prion disease of cervids, including mule deer (*Odocoileus hemionus*), whitetail deer (*Odocoileus virginianus*), Rocky Mountain elk and red deer (*Cervus elaphus* sspp.), reindeer (*Rangifer tarandus*) and moose (*Alces alces*) [1–3]. The disease has been identified in free-ranging and farmed cervids in 26 US states and 5 countries outside of the United States – most recently with novel disease foci reported across northern portions of the Scandinavian Peninsula [4–6]. New foci of the disease have been uncovered at the rate of roughly one state per year since its initial discovery outside of the original endemic zone of northern Colorado and southern Wyoming in the late 1990s.

The management of CWD in wild deer and elk herds has proven difficult, if not impossible, since being found in free-ranging mule deer and elk four decades ago [7]. Currently, the state of New York is singular in its effective surveillance and eradication of a small focus of CWD in whitetail deer, however the disease is fairly well established in wild cervid populations in neighbouring Pennsylvania and has recently been reported in captive facilities in neighbouring Ohio and Quebec [8–10]. In 2018, Norway attempted to significantly reduce the population size of reindeer in one area where CWD was discovered [11], however the discovery of the disease in neighbouring Finland and Sweden suggests the endemic area may be larger than initially believed.

Discovery of CWD on privately-owned cervid farms results in immediate quarantine of the premises and almost inevitably herd depopulation, with farmers compensated by federal and/or state indemnity funds [12]. Very rarely, property owners forgo indemnity and depopulation, and attempt to manage their herds under a strict quarantine [13]. These unique situations provide for the limited development of small-scale management strategies in controlled populations that could someday be applied to the management of larger, free-ranging herds. This manuscript describes one such property, a herd of over 570 elk maintained on 3500 acres of fenced habitat in northwestern Colorado.

We began the study hopeful that CWD could be effectively managed using a combination of early detection using rectal biopsies and the culling of CWD-positive animals, though ultimately the financial strain of managing a large CWD positive elk herd under near free-ranging conditions became too difficult for the herd owners to bare. During the second half of the project, we attempted to identify cows with specific alleles for the elk prion gene, *PRNP*, known to correlate with lower CWD susceptibility [14], including both 132LL homozygous and 132ML heterozygous animals, and separate them from the rest of the herd. Our expectations were that these cows would be selectively bred to increase the frequency of less susceptible alleles in the herd at large, however they remained unbred as management objectives evolved into a depopulation effort. Taking advantage of data available, we explored the relationships between CWD, elk genotype, and fitness, and investigated correlations between both CWD status and *PRNP* genotype with pregnancy, body condition score (BCS) and calf survival. Throughout the study, we continued to compare the performance of a prion amplification assay, RT-QuIC, with conventional immunohistochemistry in the antemortem detection of CWD, ultimately correlating our findings to post-mortem testing and survival.

Despite fluctuations in management objectives over the course of the study, we report a number of important findings. First, we found that RT-QuIC assay was significantly more sensitive than conventional IHC in the antemortem detection of CWD positive elk in recto-anal mucosa-associated lymphoid tissue (RAMALT), by a factor of nearly 50%. As an antemortem sample in elk, however, RAMALT remained clearly imperfect compared to post-mortem testing of the brainstem and retropharyngeal lymph nodes. Second, we found that CWD negative elk were 3.6 times more likely to survive year over year than those in which CWD was detected antemortem. Third, we report that neither CWD status nor *PRNP* genotype correlated with lower pregnancy rates in elk, and that *PRNP* genotype did not have an obvious effect on calf survival in the first and second year of life. Finally, while no correlation was found between body condition score and either sex or *PRNP* genotype, animals testing positive for CWD antemortem were found to be in generally poorer body condition than those testing negative antemortem.

Over the course of this multi-year longitudinal study, we learned that – as is frequently observed with CWD management in wild populations – the involvement and full cooperation of herd owners (in the case of wild cervids, the public at large) is critical for even the slightest chance of managing the disease. The demise of this particular herd seemed almost inevitable, however, regardless of our management directives. Despite this initial failure, our hope is that this primary effort of managing CWD in farmed elk may serve as a learning tool for future management efforts for this devastating disease in either privately-owned or wild herds.

## Materials and methods

### Ethics statement

All animals in this study were handled humanely in accordance with Midwestern University’s Animal Care and Use Committee, approval #2814. Animals selected for euthanasia were humanely euthanized in accordance with guidelines issued by the American Veterinary Medical Association.

### Study area and population

Details on the study population and property have been reported previously [13]. Briefly, the study was conducted in a 3500-acre (14km^2^) fenced-in area of private land in Colorado with features similar to other areas of Colorado with endemic CWD. Chronic wasting disease was first reported on the property in 2004, and prevalence rose steadily over the ensuing years. In the first year of this study (2016), prevalence was approximately 15% in adult elk based on antemortem and post-mortem testing (where available). Prevalence in mule deer and elk outside the fence is unknown, although it is presumed to be greater than 5-10% [15,16]. Future proposed testing requirements may provide more insight into current CWD prevalence in the area [17]. The herd initially consisted of over 450 animals in the first year of the project, declining to 400 and eventually 150 in the second and third years of the project (2017 and 2018), respectively. The decline was a combined result of depopulation efforts targeting CWD-positive animals, CWD-associated and unassociated animal deaths, and heavy hunting pressure as the herd was passively depopulated in the second and third years of the study. Animals were handled once yearly in the late winter, as they were run through a modern handling facility for inventory, sample collection, and routine medical treatments. Animals were identified using ear tags, RFID chips, and tattoos. In the winter of 2017, twelve 132LL and thirteen 132ML cow elk were separated out into a 100-acre pasture to be bred to bull elk with 132LL alleles; those cows were never bred and remained fallow in 2018.

### Study design

Details on the study design have been reported previously [13]. In short, a range of empirical data and clinical samples were collected from both adult and calf elk during the annual winter inventory. Empirical data included body condition scoring on all animals and pregnancy evaluation of adult cows over two years of age; both data sets were collected by an experienced large animal veterinarian. Clinical samples collected from adult animals included blood, faeces and rectal biopsies as described below. In calves less than one year of age, only blood samples were collected for genetic analysis. Adult cows were held in a separate pen after sampling to allow for the targeted culling of CWD positive individuals. Adult bulls were turned out into a 500-acre bull pen regardless of CWD status, and all calves were released onto a 500-acre spring pasture. Cows positive for CWD were humanely euthanized with a range of samples collected during necropsy, including conventional diagnostic samples. All CWD negative cows were released onto the spring pasture with the calves except for the aforementioned 132ML and 132LL cows.

### Antemortem and post-mortem sampling and data collection

During sample collection, animals were restrained using a conventional large animal squeeze chute. Blood was collected from the jugular vein and placed into a tube containing ethylenediaminetetraacetic acid tetrasodium salt (EDTA) preservative. Faeces were collected using manual evacuation, with several pellets placed in storage bags for ancillary testing [18]. Rectal biopsies were collected using sterile, single use instruments by removing a 2cm^2^ piece of mucosa from the wall of the rectum, approximately 2cm proximal to the mucocutaneous junction of the anus as described previously [19,20]. A 0.5cm^2^ subsection of this biopsy was placed into a 1.5ml microcentrifuge tube and frozen for RT-QuIC analysis, with the remainder placed into a histology cassette and preserved in 10% neutral buffered formalin (NBF) for IHC analysis.

Body condition scoring was conducted by both visual analysis and palpation of the ribs, spine, hip bones, rump, tail head and belly of the animals according to published guidelines [21]. Scores ranged from 1 to 5, though observed scores rarely exceeded 3. Pregnancy was assessed by rectal palpation, with animals typically in the late second trimester of pregnancy.

Post-Mortem samples were collected immediately after euthanasia, and where available from animals that died in the field (e.g. those hunted or dying of natural causes). At a minimum, the obex region of the brainstem, the medial retropharyngeal lymph nodes, and tonsil were collected, when available, and stored in 10% NBF for post-mortem assessment of CWD status.

### Immunohistochemistry (IHC)

Rectal biopsies and post-mortem samples were evaluated for PrP^res^-specific immunostaining as described previously, blindly and without information on the index test (RT-QuIC) results [13]. Briefly, IHC staining for PrP^res^ was performed using the primary antibody Anti-prion 99 (Ventana Medical Systems, Tucson, AZ) and counterstained with haematoxylin. Positive and negative controls were included in each analysis. Biopsies were considered positive if at least one follicle exhibited PrP^res^-specific staining. In cases where biopsies had five or fewer follicles, samples were classified as ‘insufficient follicles’ unless CWD-specific immunostaining was observed.

### Statement of transparency on RT-QuIC testing

Over the course of testing in the third year of study, a single amplification plate was discarded due to equipment malfunction. Cumulatively, 847 patient samples were run on 31 experimental plates over the course of the entire study; of these, three plates were repeated when positive controls failed to amplify, one was repeated due to a single negative control replicate amplifying, and two were repeated due to equipment malfunction.

### Real time quaking-induced conversion assay (RT-QuIC)

Rectal biopsy subsections were prepared as 10% homogenates in phosphate-buffered saline (PBS) and analysed for PrP^res^ conversion activity as described previously [6]. Amplification was performed using a truncated form of recombinant Syrian hamster PrP (SHrPrP, residues 90–231) as a conversion substrate. The details on reaction conditions are described elsewhere [14]. Test samples were analysed blindly, without information on the reference test (IHC) results, and were each repeated in triplicate on a single plate. Positive and negative controls (consisting of CWD-positive brain and biopsy homogenates from three known CWD-negative whitetail deer, respectively) were included in each analysis in triplicate. An unspiked ‘water negative’ negative control was also included in triplicate in each experiment. Reactions were prepared in black 96-well, optical bottom plates, which were then sealed and incubated in a BMG Labtech Polarstar^TM^ fluorimeter. Amplification parameters and relative fluorescence monitoring are described elsewhere.

Criteria for identification of positive samples was determined *a priori* and was consistent with previous studies in our laboratories [13,19,22,23]. Briefly, a replicate well was considered positive when the relative fluorescence crossed a pre-defined threshold, calculated as ten standard deviations above the mean fluorescence of all sample wells – positive controls, negative controls, and test samples – across amplification cycles 2–8. Positive samples were those which crossed the threshold in ≥2 out of 3 replicates; animals with 1 of 3 replicates positive were considered ‘suspects.’ Plates were disqualified if positive controls failed to amplify, or if amplification was observed in any of the various negative control replicates.

### PRNP analyses

Nucleic acids were extracted from whole blood samples preserved in EDTA using a conventional DNA extraction kit (ThermoFisher, USA). The *PRNP* gene sequence was amplified by conventional PCR and sequenced as previously described [13,22,23]. Sequences were viewed using Geneious software version 10.2 (www.Geneious.com) with specific single nucleotide polymorphisms at position 403 of the elk *PRNP* gene used to classify animals into 132MM, ML, or LL genotypes. Several other nucleotide polymorphisms were observed, though not considered for the present analysis.

### Data analysis

Statistical analysis of several components of the data set were analysed using GraphPad Prism 8.0 software. A two-tailed Fisher’s exact test was used to compare the number of CWD-positive animals identified by IHC and RT-QuIC, the *PRNP* genotype of those animals which were RT-QuIC positive and either IHC positive or IHC negative, the prevalence of CWD among different *PRNP* genotypes, the return rates of infected and uninfected animals, and the pregnancy status of animals with various CWD and *PRNP* genotypes. A Student’s t-test was used to compare the average age of RT-QuIC positive animals which were either IHC positive or IHC negative, as well as the average age of first detection in elk with different *PRNP* alleles.

To analyse the relationships between body condition score, *PRNP* genotype, sex, and CWD status, a three-factor ANOVA analysis with regression was conducted using the Real Statistics Resource Pack software (Release 6.2) [24].

## Results

### Antemortem detection of CWD in RAMALT by immunohistochemistry and RT-QuIC

**Figure 1.**
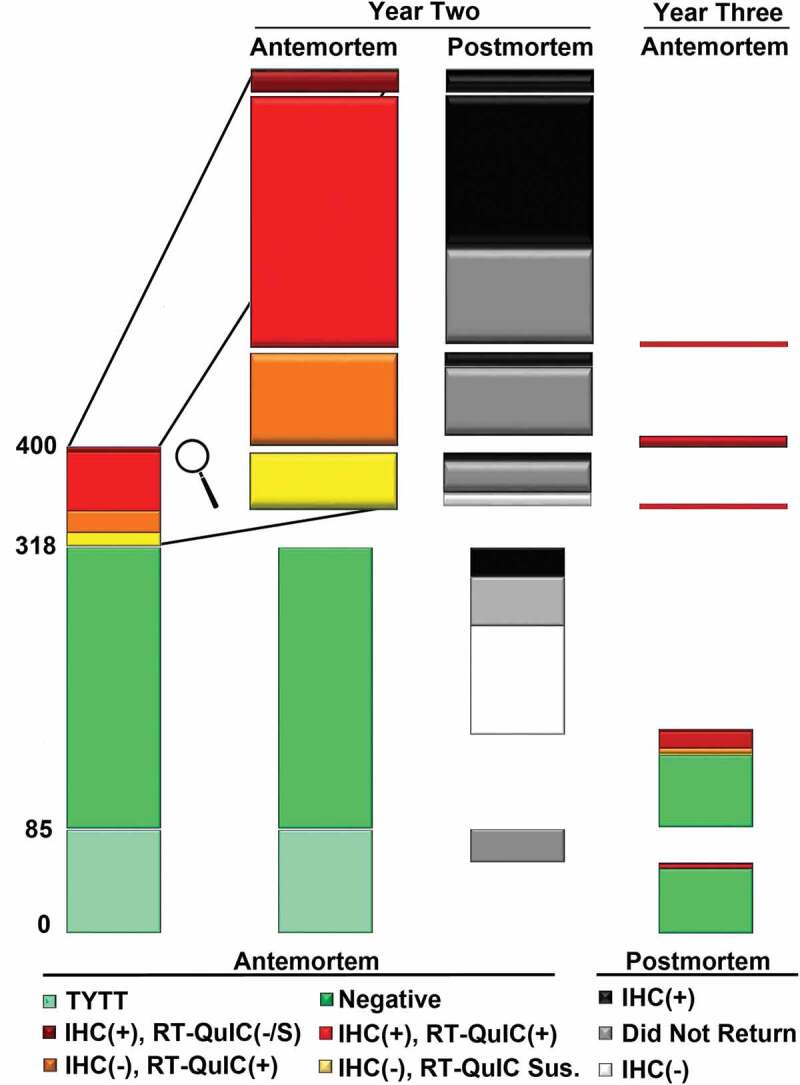
Overview of antemortem testing and post-mortem findings (where available) from study years two and three. Elk were grouped into categories based on testing results, including: (1) “too young to test’ (TYTT), (2) negative by both immunohistochemistry (IHC) and real-time quaking induced conversion (RT-QuIC), (3) IHC positive and RT-QuIC negative or suspect, (4) IHC positive and RT-QuIC positive, (5) IHC negative and RT-QuIC positive, and (6) IHC negative and RT-QuIC suspect. No animals testing positive by either antemortem assay were found to be negative post-mortem, with many not returning for sampling the following year.

In the third year of the study, 141 animals had RAMALT biopsies collected antemortem and tested for CWD, with twenty-nine found to be CWD positive (20.6%). Of those, three were positive by IHC only (one of which was considered suspect by RT-QuIC), twenty were positive by both IHC and RT-QuIC, and six were positive by RT-QuIC only. Two IHC negative elk were considered RT-QuIC suspects, with the remaining 110 animals negative by both assays antemortem. (Table 1 and Figure 1)

**Table 1.** Summary of antemortem and post-mortem testing by assay and result. Chronic wasting disease infection status in elk was categorized based on immunohistochemistry (IHC) and real time quaking-induced conversion (RT-QuIC) results from rectal biopsies. Where available, post-mortem samples were collected from animals in the second year of the study either following euthanasia or from animals harvested in the field, to confirm their CWD status. Animals from the second year of sampling which were not harvested, and did not return for the third year of the study, were presumed to have died in the field, with no sample available (NSA) for post-mortem testing. Final post-mortem test results from the third year of the study were typically not available.

Study Year Two (2017)						Study Year Three (2018)					
			Post-Mortem Test Result				Antemortem Test Result				
Antemortem Test Result	Sex	Number	CWD(+)	CWD(-)	NSA	Returned	IHC(+) only	IHC(+), RT-QuIC(+)	RT-QuIC(+) only	IHC(-), RT-QuIC Suspect	(IHC(-), RT-QuIC(-)
IHC(+), RT-QuIC(-)	Bull	0	0	0	0	0					
	Cow	2	2	0	0	0					
IHC(+), RT-QuIC Suspect	Bull	0	0	0	0	0					
	Cow	2	2	0	0	0					
IHC(+), RT-QuIC(+)	Bull	19	1	0	17	1	0	1	0	0	0
	Cow	30	29	0	1	0					
IHC(-), RT-QuIC(+)	Bull	8	2	0	6	0					
	Cow	10	1	0	7	2	0	1	1	0	0
IHC(-), RT-QuIC Suspect	Bull	6	1	1	3	1	0	1	0	0	0
	Cow	5	1	1	3	0					
IHC(-), RT-QuIC(-)	Bull	81	3	20	15	43	2	7	0	1	33
	Cow	152	21	70	25	36	0	7	4	1	24
Untested Calves	Bull	37	0	0	13	24	0	0	0	0	24
	Cow	48	1	8	5	34	1	3	1	0	29
Total	Bull	151	7	21	54	69	2	9	0	1	57
	Cow	249	57	79	41	72	1	11	5	1	53

Over the course of the three-year study [13], 100 unique animals were identified as CWD positive by antemortem IHC of RAMALT, and of these 71 were confirmed positive by post-mortem IHC; the remaining 29 perished in the field untested. Of the 147 unique individuals identified as RT-QuIC positive across the three-year period, 75 were confirmed positive by post-mortem IHC; the remaining 72 animals perishing in the field. (Figure 1 and Supplementary Figure 1) The large discrepancies between post-mortem sample availability is primarily explained by the management practices, where all cows identified as IHC positive antemortem were euthanized, while the majority of the animals which were IHC negative yet RT-QuIC positive were released. Post-Mortem testing uncovered 38 additional CWD positive animals considered negative by both assays, with 9 testing positive in the RLN only, and 29 testing positive in both obex and RLN several months to a full year after initial antemortem screening. The cumulative number of positive animals identified by antemortem IHC or RT-QuIC, or post-mortem IHC totalled 193 animals. Taken collectively, our findings suggest that the antemortem sensitivity of IHC on RAMALT is roughly 52%, with RT-QuIC identifying significantly more positives with a sensitivity of 76% (p < 0.01). Determining specificity of the IHC and RT-QuIC assays given the study design is more problematic, however we can report that no animal testing positive by either assay antemortem was later found to be CWD negative by post-mortem testing. As such, our initial supposition is that the specificity of both assays is 100%.

### Factors affecting CWD detection by IHC

To this point, three factors have been implicated to explain why CWD positive elk or deer may not be positive by IHC on antemortem RAMALT testing: low follicle counts, *PRNP* genotype, and stage of infection [19,23,25]. Although we were not able to assess disease stage effectively in the present study, we did have data on several other metrics available to examine why RT-QuIC positive samples may be IHC negative. A significantly lower number of follicles was observed in RT-QuIC positive, IHC negative samples compared to RT-QuIC positive, IHC positive samples, and has been reported elsewhere (23.2 vs. 32.3, respectively), though were in both cases generally higher than the five follicle target for greatest sensitivity [26,27]. Over the course of the study, fifteen samples that were RT-QuIC positive had five or fewer follicles on IHC evaluation [25,28], suggesting that high numbers of follicles may not be required for RT-QuIC amplification. Other factors we sought to consider here were animal age and *PRNP* genotype.

While the average age of animals with RT-QuIC positive, IHC negative biopsies across the three-year study period was not significantly different from those with RT-QuIC positive, IHC positive biopsies (5.77 vs. 4.98, p = 0.1145), there was a trend towards older, RT-QuIC positive animals being IHC negative. Interestingly, an RT-QuIC positive animal which was IHC negative was more likely to have a 132ML genotype than one which was positive by both assays across the three-year study period (p = 0.017). Among the RT-QuIC positive, IHC negative samples, there were twenty-seven 132MM individuals and twenty-nine 132ML animals; among those which were RT-QuIC positive and IHC positive, there were sixty-seven 132MM individuals and thirty-one 132ML animals. Taken together, these latter two points hint that *PRNP* genotype, a variable shown to affect CWD progression in cervids, may have an impact on antemortem testing sensitivity – potentially favouring the RT-QuIC assay.

### Prevalence of CWD among elk with different PRNP backgrounds

Between sample collection periods in years two and three, a total of 164 animals were euthanized or hunted and tested for CWD. An additional 95 were lost in the field, thus their post-mortem CWD status could not be determined. Among elk with the 132MM genotype, a total of 35/56 animals (62.5%) were found to be CWD positive by antemortem testing, post-mortem testing, or both, while in animals identified as 132ML heterozygous, 23/98 (23.5%) were found to be CWD positive. Five of twelve 132LL homozygous animals (41.7%) were found to be CWD positive. (Table 2)

**Table 2.** Third year summary of antemortem and post-mortem testing by *PRNP* genotype. Immunohistochemistry (IHC) and real time quaking-induced conversion (RT-QuIC) were used to evaluate rectal biopsies from ranched elk for evidence of chronic wasting disease (CWD). Post-Mortem samples were collected either following euthanasia or when available from animals harvested in the field, to confirm their CWD status. Animals from the second year of sampling which were not harvested, and did not return for the third year of the study, were presumed to have died in the field, with no sample available (NSA) for post-mortem testing. Final post-mortem test results from the third year of the study were typically not available.

		Number Tested			Antemortem test results					Post-Mortem IHC results			
Study Period	132 *PRNP*	Bulls	Cows	Untested Calves	IHC+Only	IHC(+), RT-QuIC(+)	RT-QuIC(+)Only	IHC(-), RT-QuIC Suspect	IHC(-), RT-QuIC(-)	CWD(+)	CWD(-)	NSA	Returned
Year 2	MM	38	74	45	2	36	5	3	66	35	21	40	61
	ML	63	115	30	0	14	11	8	145	23	75	45	68
	LL	13	12	10	0	1	0	0	24	5	7	8	15
Year 3	MM	30	31	1	1	12	5	1	42	17	16	29	0
	ML	29	36	3	1	7	1	1	55	12	21	35	0
	LL	10	5	5	1	1	0	0	13	1	2	17	0

In sample collection year three, a total of 141 adult elk were tested antemortem for CWD. Among the 132MM homozygous animals tested, 18/61 (29.5%) were positive by either IHC or RT-QuIC, or both; one animal was considered an RT-QuIC suspect, with the remaining testing negative by both IHC and RT-QuIC. Testing of 132ML heterozygous animals identified 9/65 animals as CWD positive (13.8%); an additional animal was considered suspect by RT-QuIC, and the remaining 55 animals were RT-QuIC negative. Finally, two of fifteen 132LL homozygous animals (13.3%) were found positive by RT-

QuIC and/or IHC; the remaining eighteen animals were all negative by both assays. (Table 2)

Throughout the study, 565 individual elk, including both adults and calves, were sampled during the course of annual inventory [13]. Among 232 animals identified as 132MM homozygous, 112 were found to be CWD positive either ante- or post-mortem (48.3%). Seventy of 283 animals with the 132ML genotype (24.7%) were found to be CWD positive, while seven of 50 132LL homozygous animals were positive for CWD (14%). Animals homozygous for the 132M allele were nearly twice as likely to be identified as CWD positive compared to their 132ML counterparts (relative risk: 1.95, p < 0.001; 95% confidence interval 1.53–2.49). Animals with the 132MM genotype also faced a risk of nearly 3.5 times that of 132LL homozygous genotype (relative risk: 3.45, p < 0.001, 95% confidence interval 1.71–6.94). In turn, 132ML heterozygous animals were not significantly more likely to be found CWD positive than 132LL homozygous animals, although there was a trend to indicate they may be (risk ratio: 1.59, p = 0.19; 95% confidence interval 0.7815–3.24).

### Age at initial CWD detection among elk with different PRNP backgrounds

In an effort to better understand the dynamics of CWD infections in elk with varying *PRNP* backgrounds, we examined the age of initial detection of CWD, by either antemortem or post-mortem testing, in elk with 132MM, 132ML, and 132LL genotypes across all years of the study.

**Figure 2.**
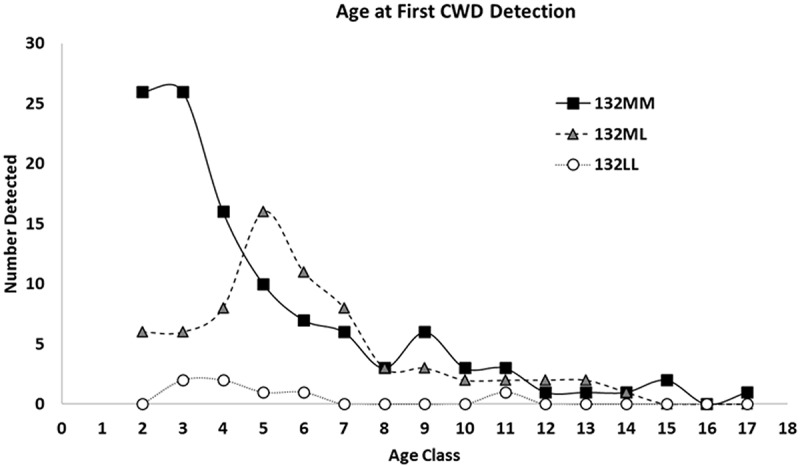
Age of first detection of CWD infection in elk, based on combined antemortem and post-mortem testing data. Antemortem testing combined information collected from both the real-time quaking induced conversion assay (RT-QuIC) and immunohistochemistry (IHC), while post-mortem data was based on IHC testing. Genotypes were confirmed by sequencing of the *PRNP* gene.

The average age of first detection of CWD in 132MM individuals was 4.98 years, while the average age among 132ML animals was 6.01 years (p = 0.039). This difference was most apparent among males, where 132MM bulls were identified as CWD positive at 3.60 years of age, on average, compared to 132ML bulls where the average age of first detection was 4.79 years (p = 0.01). The difference between age at first detection in cows, however was not significant (5.76 vs. 6.47 for 132MM and 132ML cows, respectively; p = 0.27). The average age of detection in 132LL animals was not significantly different from that of 132ML animals, in either bulls or cows. (Figure 2 and Supplementary Table 1)

### Return rate of CWD positive animals

In the 2017 sampling period, 315 animals were tested for CWD antemortem. Of those, 71 tested positive by IHC, RT-QuIC, or both. Thirty-four infected animals (thirty-three cows and one bull) were euthanized and confirmed CWD positive post-mortem, with the remaining thirty-seven animals (Twenty-six bulls and eleven cows) released back onto the property. Of those animals which were released, four were harvested in the fall of 2017 and were found to be CWD positive post-mortem. Three of the remaining thirty (10%) returned for the 2018 sampling period, the remainder were lost in the field and went untested. All three were 132ML heterozygous animals, each positive again on antemortem testing in year three. In contrast, 120 of the 329 animals negative by antemortem testing were harvested in the fall of 2017, with 26 found to be CWD positive (21.7%). Of the remaining 209 animals, 141 returned for the 2018 sampling period (67.5%), with the remaining animals presumed lost in the field, untested. (Table 1 and Figure 1)

Over the course of the entire study, four of forty-nine CWD positive 132MM animals released back onto the property (8.2%) returned for a second year of sampling. Nine of thirty-three 132ML animals positive for CWD returned for a second year of sampling (27%), a return rate that was significantly greater than that of CWD positive 132MM animals (risk ratio: 3.34, p = 0.03). The lone 132LL cow identified during the course of antemortem testing in year two did not return for sampling in year three. Cumulatively, just 13 of 82 animals identified as CWD positive and released onto the property returned the following year (15.9%). These low rates of yearly return are in stark contrast to the cumulative return rate for CWD negative animals. For animals homozygous for the 132M allele, 107/144 returned in year two, and 28/69 returned in year three (63% overall). For 132ML heterozygous animals, 170/221 returned in year two, with 62/181 returning in year three (58% overall). Twenty-five of thirty-five animals homozygous for the 132L allele returned in year two, with fifteen of thirty-five returning in year three (57% overall). Cumulatively, 60% of animals negative for CWD returned the following year – a yearly return rate nearly over 3.5 times that of CWD positive animals (risk ratio: 3.62, p < 0.001; 95% confidence interval 1.96–6.69).

### Pregnancy rates in animals with varying PRNP backgrounds and CWD status

Previous studies have found variable influences of *PRNP* polymorphisms on fertility [29,30], while one recent report has evaluated fertility rates of mule deer in an area with endemic CWD and found no relationship between infection status and pregnancy [31]. Over the course of two sampling years, we evaluated late-term pregnancy in 251 cow elk over two years of age to compare the pregnancy rates across various *PRNP* genotypes, as well as to CWD status.

Pregnancy rates did not differ significantly between 132MM and 132ML cows across both years of the study (61% and 68% respectively, *P* = 0.27). Pregnancy rates were significantly higher in 132ML animals compared to 132LL animals (33%, *P* < 0.05), however it should be noted that the separation of 12 adult 132LL cows in the second study year resulted in just five 132LL cows, all 2-year-olds, for inclusion in our pregnancy evaluation in year three. (Table 3) Interestingly, cows positive for CWD were significantly more likely to be found pregnant than CWD negative cows (73.4% vs. 59.1%, respectively, *P* < 0.05). (Table 3)

**Table 3.** Pregnancy status among cow elk of various *PRNP* genotypes (3a) and CWD status (3b). Pregnancy was determined by rectal palpation, with *PRNP* genotype confirmed by sequencing and CWD status confirmed by antemortem rectal biopsy testing.

	132MM		132ML		132LL	
Study Period	Pregnant	Open	Pregnant	Open	Pregnant	Open
Year 2	46	21	75	30	5	5
Year 3	13	17	19	14	0	5
Cumulative	59	38	94	44	5	10

	CWD positive		CWD Negative	
Study Period	Pregnant	Open	Pregnant	Open
Year 2	46	17	80	39
Year 3	12	4	20	32
Cumulative	58	21	101	71

### Body condition scores among elk with different PRNP backgrounds and CWD status

In a previous study, cervids with *PRNP* alleles found to be less susceptible to CWD were anecdotally characterized as having ‘less than optimal’ body condition [32]. Meanwhile, poor body condition is often associated with animals terminally affected with CWD [1]. In an effort to determine whether body condition scores correlated with either *PRNP* genotype or CWD status, we evaluated BCS for 354 animals in year two and 150 animals in year three of the study.

Using three-factor ANOVA analysis, no correlation was observed between BCS and either sex or genotype. Perhaps not surprisingly, a correlation was observed between CWD status and BCS, with CWD positive animals generally scoring lower than those found negative by antemortem testing. (Table 4 and Supplementary Table 2)

**Table 4.** **Correlation of sex, CWD status, and Genotype with Body Condition Scores**. Body condition scores were collected from elk by manual palpation during sampling in Study Years 2 and 3, and were correlated to sex, CWD status, and *PRNP* genotype using a three-way ANOVA analysis with regression. A significant correlation was observed between CWD status and body condition score.

Factor(s) Considered	p-value
Sex	0.742046
CWD Status	0.008674
Genotype	0.079612
Sex and CWD Status	0.780121
Sex and Genotype	0.670946
CWD Status and Genotype	0.078185
Sex, CWD Status, and Genotype	0.708582

### Annual survival of young elk with different PRNP backgrounds

The same study referenced above has also suggested, anecdotally, that cervids with less susceptible *PRNP* alleles may be faced with poor young recruitment [32]. We therefore considered the annual return rate of calves and yearling elk in an effort to identify any genotype-specific correlations.

**Figure 3.**
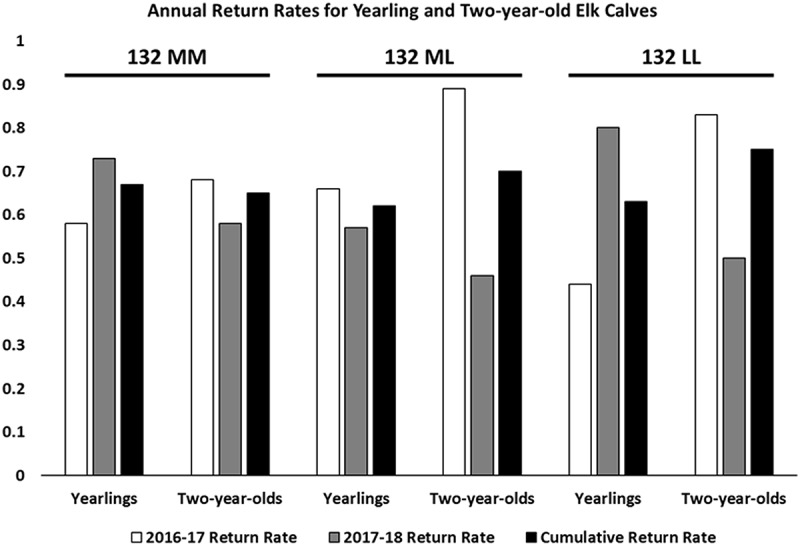
Annual return rates for yearling and two-year-old elk calves. Return rates of young elk were calculated based on animals present at inventory across the 2016–2018 sampling periods. Genotypes were confirmed by sequencing the *PRNP* gene.

We found that calf return rates were not significantly different across elk *PRNP* genotypes, with 51 of 76 132MM, 42 of 68 132ML, and 12 of 19 132LL calves returning for a second year of sampling. The same held true for yearling elk, where 49 of 75 132MM, 45 of 64 132ML, and 12 of 16 132LL animals returned in the following year for sampling. (Figure 3)

## Discussion

While reports on the management of chronic wasting disease in wild deer and elk are many and varied [33–43], rare is the case presented for managing the disease in farmed cervids. Almost without exception, farmed cervids are immediately placed under quarantine and eventually depopulated when CWD is discovered on site [12]. This manuscript reports our efforts to manage CWD in a large elk herd, in a controlled setting with endemic CWD, through the use of annual live animal testing and targeted culling of CWD positive cows. Although the herd owners were presented with additional management directives, including culling of CWD positive bulls and those animals positive by an amplification assay (RT-QuIC), they were not implemented due to concern regarding its potential impact on hunting revenue. Ultimately, we could not completely evaluate our management practices, as the herd was slowly depopulated after the final sampling period due to the financial burden brought by the disease.

Over the three-year study, more than 550 animals were sampled, including calves, bulls, and cows – many on multiple occasions. The study design provided information on several aspects of this disease that had not been explored or reported previously in this species, including the longitudinal evaluation of elk by RT-QuIC on samples collected antemortem. Importantly, we found that the RT-QuIC assay provided a significant improvement in sensitivity over conventional IHC in this study, identifying positive animals irrespective of either follicle count or *PRNP* genotype – two factors known to affect RAMALT IHC sensitivity [25–27]. Our sensitivity estimates for these two assays in elk (52% for IHC and 76% for RT-QuIC) were comparable to those in a previous study in whitetail deer [23]. A more limited study in elk found that RT-QuIC and IHC sensitivities were similar to one another (77%, 21); however, conditions were ideal for immunohistochemical detection in the farmed herd reported in that study – including high biopsy follicle counts, a high frequency of the 132M allele, and many of the animals in advanced stages of disease. Ultimately, elk in the present study identified as CWD positive by either the RT-QuIC assay or IHC were 3.6 times more likely to die in the field over the course of a year than their CWD negative cohorts. These dramatically different survival patterns are largely comparable to those reported for whitetail and mule deer in CWD endemic areas [31,44]. The few positive elk that did return were nearly 3.5 times as likely to have the 132ML genotype and were inevitably positive again antemortem, and no animal diagnosed as having CWD was found to have survived a second year.

Our study also allowed us to explore the relationship between *PRNP* genotype and (1) age at first detection of CWD infection, (2) pregnancy rates and (3) year over year calf and yearling survival. In addition to confirming reduced susceptibility in elk with the 132ML and 132LL genotypes, nearly one quarter that of 132MM elk, we report that animals with the 132ML genotype were found to be CWD positive a full year later, on average, than their 132MM counterparts. Pregnancy rates between 132MM and 132ML cows were similar, though elk pregnancy rates can be highly variable and affected by factors outside of the scope of the present study, including population density [45]. The relatively low numbers of 132LL animals made each of these comparisons difficult – especially when considering pregnancy rates and the unfortunate fact that 132LL cows were not allowed to be bred between the second and third years of sampling. Annual calf and yearling survival rates in 132MM, 132ML, and 132LL animals were similar, although we could not assess survival for the first nine months of life due to the timing of sample collections, an admitted limitation in our assessment.

Lastly, we were able to evaluate the relationships between body condition scores (BCS) and both *PRNP* genotype and CWD status. Using a three-factor ANOVA approach, we found no significant correlation between BCS and sex; and although the differences observed in BCS across genotypes were not significant, there was a trend towards higher body condition scores in 132ML animals that may warrant further investigation. Perhaps not surprisingly, a correlation was observed between CWD status and body condition score – with CWD positive animals generally scoring lower. Although the differences were significant, it is likely that BCS may remain an imperfect factor in the clinical differentiation of CWD positive and negative individuals.

Taken together, our findings provide insight into an important question that has been posed regarding CWD and less susceptible *PRNP* alleles: whether these rarer alleles are somehow associated with an evolutionary fitness disadvantage [32]. We found no evidence over the course of the project to suggest that 132ML elk had anything but a higher level of fitness than 132MM animals, especially in the face of a high level of endemic CWD prevalence. Less data was available on 132LL elk, and further investigations are warranted to better assess their level of susceptibility and any potential fitness costs that may be associated with this genotype.

Although we were unable to fully evaluate our management goals for this particular herd, future studies on the small-scale management of CWD in closed herds may be improved by revisiting several important aspects of the present study. First, while the antemortem collection of RAMALT tissue is relatively straightforward for both those collecting samples and those being collected from, more problematic samples like tonsil biopsies may provide a higher level of sensitivity, allowing for a more effective removal of clinical and preclinically infected animals [46,47]. Secondly, stressing the complete and timely removal of all animals identified as CWD positive will be important in limiting horizontal transmission. Third, it may prove useful to more effectively implement a genetic strategy as it relates to the *PRNP* gene – through the removal of more susceptible genotypes in favour of the propagation of those found to be less susceptible. Past studies [39,48], and studies ongoing, may provide additional insight into the utility of the genetic-based management of CWD. Lastly, it will be important to adequately address prions shed into the environment. While this aspect is inherently the most difficult to address, there are several potential approaches that may show some benefit, including pasture rotation and reduction of animal density, practices which have been used to effectively reduce the prevalence of other important diseases, including prion diseases [49–52].

Despite the failures in our approach, the present study provides firm data on the relative sensitivity of the RT-QuIC assay compared to conventional immunohistochemistry of RAMALT tissues, and these findings are very likely to extend to tissues collected post-mortem. The study also presents important data points on CWD susceptibility, survival, and disease progression in animals with different *PRNP* genotypes. It is our hope that this study will serve as a resource for game biologists seeking to model the effects of CWD on elk herds. The mistakes and hurdles encountered will almost certainly allow for the improvement of CWD management strategies in farmed elk, should the opportunity arise again in the future.
