# Supplementary material for: Management of chronic wasting disease in ranched elk: conclusions from a longitudinal three-year study
Source: Prion. 2020 Feb 7;14(1):76–87. doi: 10.1080/19336896.2020.1724754 (PMC7009334; doi:10.1080/19336896.2020.1724754)
Supplement: Supplemental Material [file kprn-14-01-1724754-s001.zip › Supplementary information/Supplementary Files.docx]

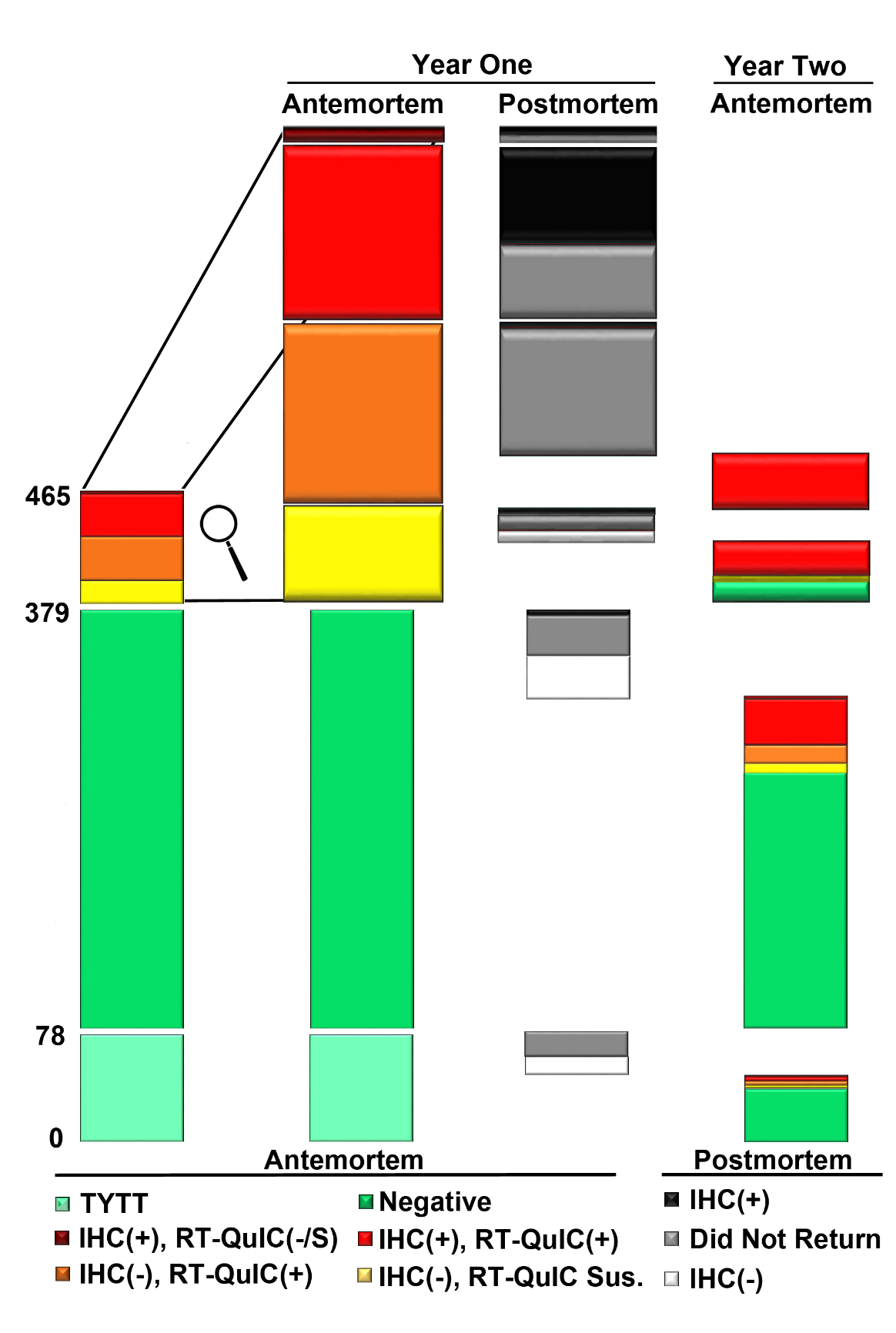


**Supplementary Figure 1.** Overview of antemortem testing and postmortem findings (where available) from study years one and two. Elk were grouped into categories based on testing results, including: 1) “too young to test’ (TYTT), 2) negative by both immunohistochemistry (IHC) and real-time quaking induced conversion (RT-QuIC), 3) IHC positive and RT-QuIC negative or suspect, 4) IHC positive and RT-QuIC positive, 5) IHC negative and RT-QuIC positive, and 6) IHC negative and RT-QuIC suspect. No animals testing positive by either antemortem assay were found to be negative postmortem, with many not returning for sampling the following year.

| Age Class | *PRNP* Genotype | | | | | |
| --- | --- | --- | --- | --- | --- | --- |
|  | 132MM | | 132ML | | 132LL | |
|  | CWD+ | | CWD(+) | | CWD(+) | |
|  | Bulls | Cows | Bulls | Cows | Bulls | Cows |
| 2 | 12 | 14 | 3 | 3 | 0 | 0 |
| 3 | 11 | 15 | 2 | 4 | 1 | 1 |
| 4 | 7 | 9 | 2 | 6 | 2 | 0 |
| 5 | 4 | 6 | 5 | 11 | 0 | 1 |
| 6 | 4 | 3 | 4 | 7 | 1 | 0 |
| 7 | 1 | 5 | 2 | 6 | 0 | 0 |
| 8 | 1 | 2 | 1 | 2 | 0 | 0 |
| 9 | 0 | 6 | 0 | 3 | 0 | 0 |
| 10 | 0 | 3 | 0 | 2 | 0 | 0 |
| 11 | 0 | 3 | 0 | 2 | 0 | 1 |
| 12 | 0 | 1 | 0 | 2 | 0 | 0 |
| 13 | 0 | 1 | 0 | 2 | 0 | 0 |
| 14 | 0 | 1 | 0 | 1 | 0 | 0 |
| 15 | 0 | 2 | 0 | 0 | 0 | 0 |
| 16 | 0 | 0 | 0 | 0 | 0 | 0 |
| 17 | 0 | 1 | 0 | 0 | 0 | 0 |

**Supplementary Table 1.** Age at first detection of CWD infection, based on combined antemortem and postmortem data. Numbers of CWD positive bulls and cows based on their age at first detection, based on data provided by antemortem immunohistochemistry or real time quaking-induced conversion, or postmortem immunohistochemistry. Genotype was confirmed by sequencing of the *PRNP* gene.

| Factor | | | Count | Mean | Variance |
| --- | --- | --- | --- | --- | --- |
| Sex | CWD Status | *PRNP* Genotype |  |  |  |
| Female |  |  | 323 | 2.386997 | 0.237967 |
| Male |  |  | 226 | 2.34292 | 0.267439 |
|  | NEG |  | 448 | 2.352679 | 0.246704 |
|  | POS |  | 101 | 2.440594 | 0.261436 |
|  |  | LL | 55 | 2.3 | 0.292593 |
|  |  | ML | 275 | 2.423636 | 0.235023 |
|  |  | MM | 219 | 2.317352 | 0.253184 |
| Female | NEG |  | 260 | 2.367308 | 0.238116 |
| Female | POS |  | 63 | 2.468254 | 0.232847 |
| Male | NEG |  | 188 | 2.332447 | 0.259209 |
| Male | POS |  | 38 | 2.394737 | 0.312945 |
| Female |  | LL | 25 | 2.24 | 0.2525 |
| Female |  | ML | 170 | 2.423529 | 0.242638 |
| Female |  | MM | 128 | 2.367188 | 0.226316 |
| Male |  | LL | 30 | 2.35 | 0.330172 |
| Male |  | ML | 105 | 2.42381 | 0.224908 |
| Male |  | MM | 91 | 2.247253 | 0.285409 |
|  | NEG | LL | 52 | 2.259615 | 0.279317 |
|  | NEG | ML | 238 | 2.418067 | 0.2306 |
|  | NEG | MM | 158 | 2.28481 | 0.249577 |
|  | POS | LL | 3 | 3 | 0 |
|  | POS | ML | 37 | 2.459459 | 0.269144 |
|  | POS | MM | 61 | 2.401639 | 0.256831 |
| Female | NEG | LL | 24 | 2.208333 | 0.237319 |
| Female | NEG | ML | 145 | 2.410345 | 0.241906 |
| Female | NEG | MM | 91 | 2.340659 | 0.227106 |
| Female | POS | LL | 1 | 3 |  |
| Female | POS | ML | 25 | 2.5 | 0.25 |
| Female | POS | MM | 37 | 2.432432 | 0.224474 |
| Male | NEG | LL | 28 | 2.303571 | 0.321098 |
| Male | NEG | ML | 93 | 2.430108 | 0.215171 |
| Male | NEG | MM | 67 | 2.208955 | 0.273858 |
| Male | POS | LL | 2 | 3 | 0 |
| Male | POS | ML | 12 | 2.375 | 0.323864 |
| Male | POS | MM | 24 | 2.354167 | 0.314764 |

**Supplementary Table 2:** Mean body condition scores, with variance, from various groupings of animals, including sex, CWD status, and *PRNP* genotype.
