# Supplementary figures and images for: Management of chronic wasting disease in ranched elk: conclusions from a longitudinal three-year study
Source: Prion. 2020 Feb 7;14(1):76–87. doi: 10.1080/19336896.2020.1724754 (PMC7009334; doi:10.1080/19336896.2020.1724754)

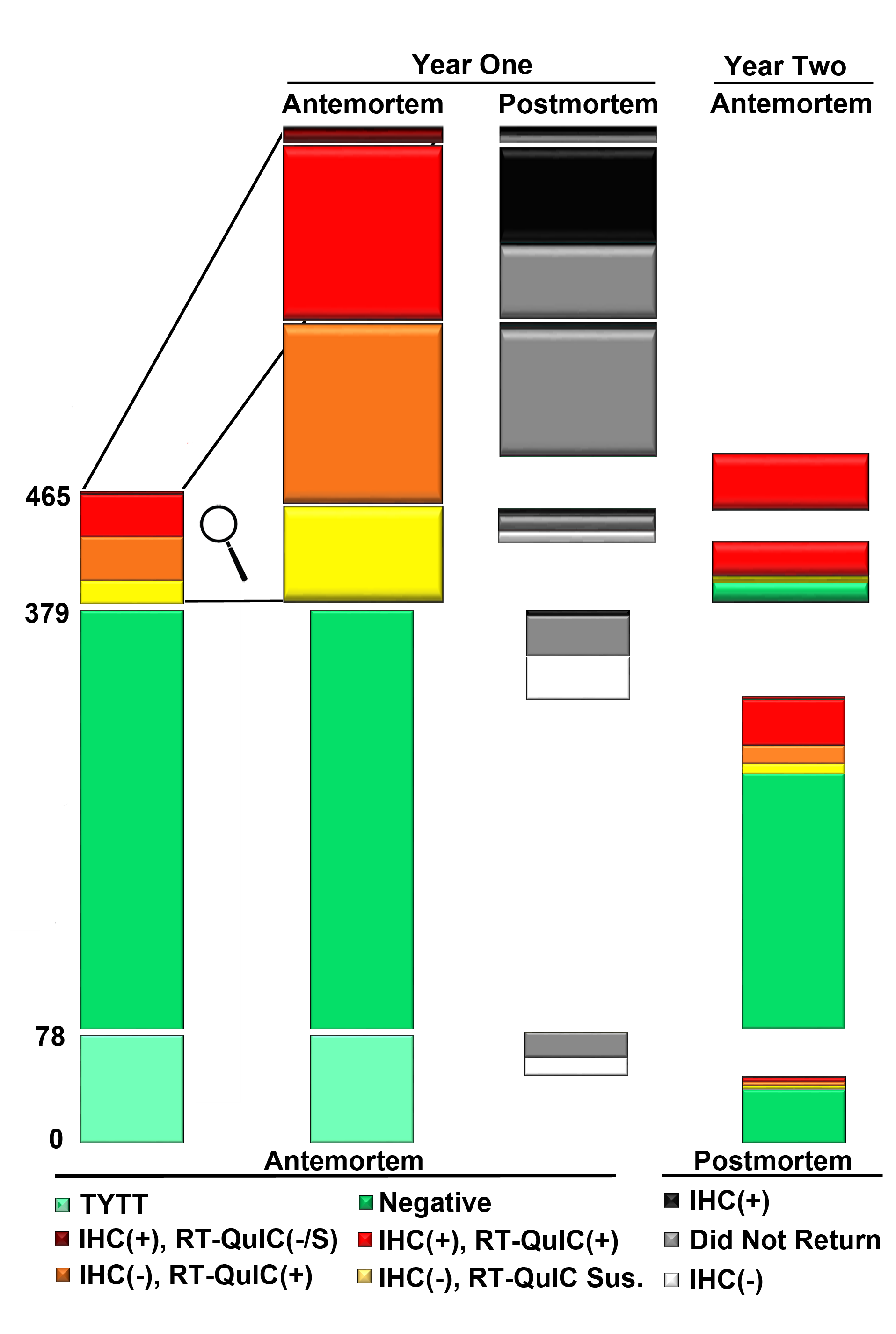

Supplement: Supplemental Material [file kprn-14-01-1724754-s001.zip › Supplementary information/E33_2SF1.tif]
